# Self-Nucleic Acid Sensing: A Novel Crucial Pathway Involved in Obesity-Mediated Metaflammation and Metabolic Syndrome

**DOI:** 10.3389/fimmu.2020.624256

**Published:** 2021-01-26

**Authors:** Amandine Ferriere, Pauline Santa, Anne Garreau, Purbita Bandopadhyay, Patrick Blanco, Dipyaman Ganguly, Vanja Sisirak

**Affiliations:** ^1^CNRS-UMR 5164, Immunoconcept, Bordeaux University, Bordeaux, France; ^2^IICB-Translational Research Unit of Excellence, Division of Cancer Biology and Inflammatory Disorders, CSIR-Indian Institute of Chemical Biology, Kolkata, India; ^3^Immunology and Immunogenetic Department, Bordeaux University Hospital, Bordeaux, France

**Keywords:** nucleic acids, obesity, metabolic syndrome, metainflammation, inflammation, nucleic acid sensing, non-alcoholic steatohepatitis

## Abstract

Obesity and overweight are a global health problem affecting almost one third of the world population. There are multiple complications associated with obesity including metabolic syndrome that commonly lead to development of type II diabetes and non-alcoholic fatty liver disease. The development of metabolic syndrome and severe complications associated with obesity is attributed to the chronic low-grade inflammation that occurs in metabolic tissues such as the liver and the white adipose tissue. In recent years, nucleic acids (mostly DNA), which accumulate systemically in obese individuals, were shown to aberrantly activate innate immune responses and thus to contribute to metabolic tissue inflammation. This minireview will focus on (i) the main sources and forms of nucleic acids that accumulate during obesity, (ii) the sensing pathways required for their detection, and (iii) the key cellular players involved in this process. Fully elucidating the role of nucleic acids in the induction of inflammation induced by obesity would promote the identification of new and long-awaited therapeutic approaches to limit obesity-mediated complications.

## Obesity-Associated Metabolic Syndrome: A Global Epidemic With an Inflammatory Origin

Over the past 4 decades the prevalence of overweight and obese individuals has continuously and substantially increased, affecting almost one-third of the world population (1). The main cause of obesity is an imbalance between consumed and burned calories (2). Obesity is associated with the development of metabolic syndrome, which is commonly defined by hypertension, hyperglycemia, excess abdominal fat, and abnormal lipidemia (1). Metabolic syndrome frequently has a “domino effect” as it leads to the development of severe diseases such as type II diabetes (T2D), non-alcoholic fatty liver disease (NAFLD), atherosclerosis, and ischemic cardiovascular disease (2). Therefore, obesity has far-reaching consequences for life expectancy, quality of life and healthcare costs (3). Treatment options for obesity are limited and include lifestyle changes that generally do not induce marked and/or sustainable weight loss and bariatric surgery, which effectively induces weight loss and reduces comorbidities but increases perioperative mortality, surgical complications and is associated with relapse (4). Furthermore, specific therapeutic targeting of either interleukin (IL-)1β (5) or tumor necrosis factor (TNF-)α (6) have shown limited success. It is therefore essential to develop new therapeutic avenues to ameliorate and prevent obesity-associated complications (7).

The main tissue affected by obesity is the white adipose tissue (WAT), which becomes hypertrophied and heavily infiltrated by immune cells that adopt a pro-inflammatory profile in response to endogenous signals (8). The resulting chronic low-grade inflammation state, also called metabolic inflammation, or “metaflammation”, plays a crucial role in the development of obesity-associated metabolic syndrome and further complications (8). In particular, macrophages (Mφ) that originate mostly from circulatory monocytes and to lesser extent from tissue resident Mφ, accumulate in the adipose tissue of obese individuals (9) and adopt an M1 pro-inflammatory phenotype (8). This switch from anti-inflammatory M2 Mφ, that are dominant in the adipose tissue of lean individuals, to M1 Mφ during obesity promotes the production of pro-inflammatory cytokines (e.g.,TNF-α and IL-1β) that can directly inhibit insulin signaling and lead to cardiovascular and metabolic complications related to obesity (8). However, targeting these cytokines has shown marginal clinical benefits for obese patients (5, 6). Furthermore, recent single-cell RNA sequencing studies have revealed a higher complexity beyond the classic M1/M2 distinction of Mφ in the WAT of obese individuals and mice (10). Therefore, the cellular and molecular mechanisms that are responsible for obesity-triggered metaflammation are not yet fully understood.

The activation of inflammatory pathways is mediated by pathogen recognition receptors (PRRs) upon sensing of exogenous pathogen associated molecular patterns (PAMPs) and endogenous damage associated molecular patterns (DAMPs). Excess nutrient intake causes an accumulation of DAMPs such as free fatty acids and cholesterol crystals but also of PAMPs such as lipopolysaccharides (LPS) originating from the intestinal microbiota in response to obesity-promoted intestinal permeability (11). These DAMPs and PAMPs were shown to contribute to obesity-mediated metaflammation by activating multiple PRRs including Toll-like receptors (TLR)2 and TLR4 and the NLR family pyrin domain containing 3 (NLRP3) inflammasome (12). It is becoming clear that obesity also induces the accumulation of nucleic acids (NA), which function similarly to DAMPs and thus activate innate immune responses (13). The source and the form of these NA are diverse and their recognition by NA sensing PRRs expressed by dendritic cells (DCs) or MΦ seems to be a key initiating event in the pathogenesis of obesity (14–16). Here, we will focus on (i) the main sources and forms of these NA, (ii) the sensing pathways involved in their detection, and (iii) the key cellular players involved in this process.

## Sources and Forms of Nucleic Acids That Accumulate During Obesity

**Figure 1:**
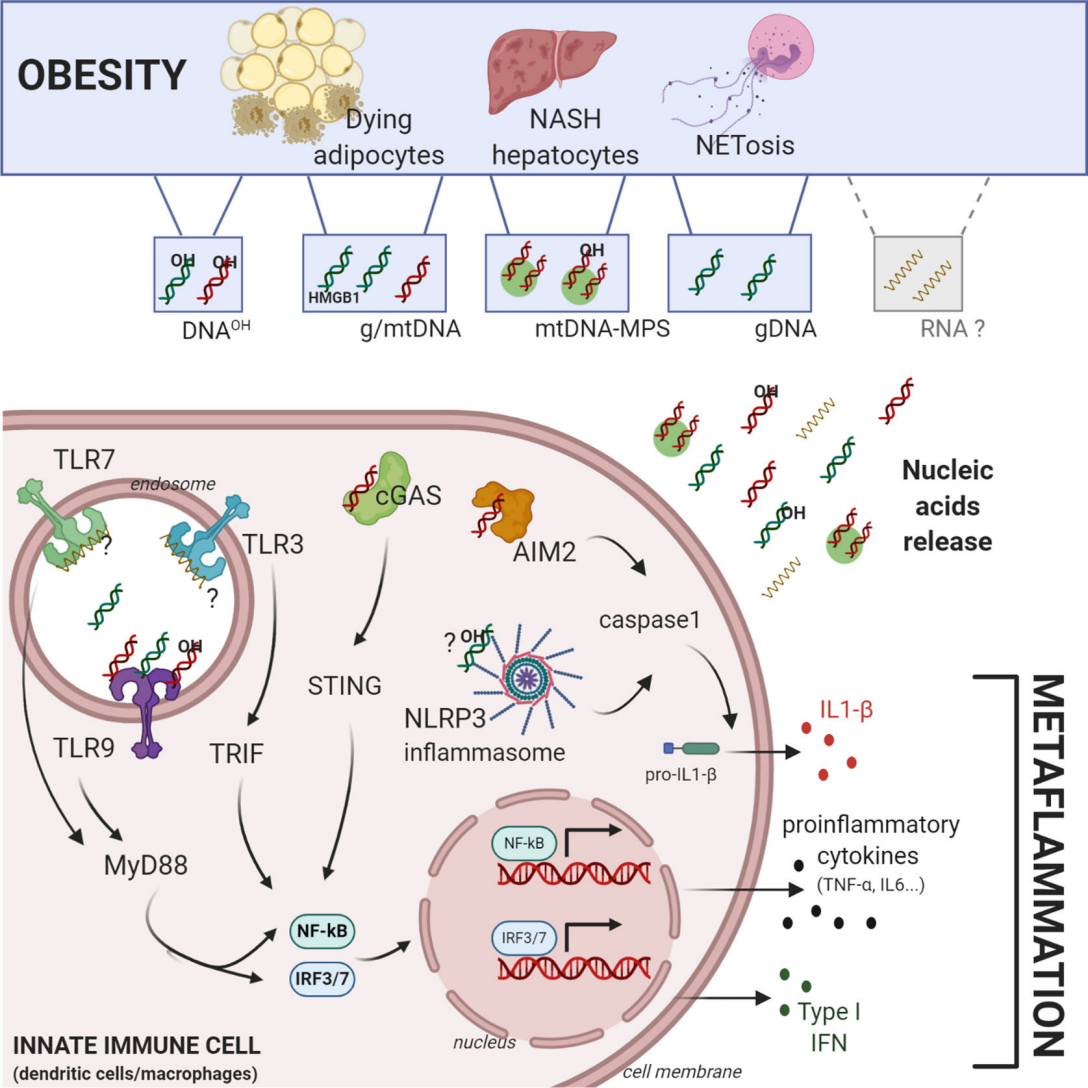
Central role of self-nucleic acids in obesity-mediated metaflammation. Obesity induces an accumulation of different sources and forms of self-nucleic acids (NA). Genomic-, mitochondrial, oxidized-DNA (OH-DNA) and potentially RNA can be released in aberrant amounts during obesity by dying adipocytes, NASH hepatocytes, or NETosis. Self-NA can activate cytosolic or endolysosomal pathogen recognition receptors in macrophages or dendritic cells such as Toll-Like-Receptor 3/7/9, cGAS-STING pathway, AIM2 and NLRP3 inflammasomes. Their activation induces *in situ* production of i) proinflammatory cytokines (IL6, TNF-α…) through the activation of NF-ΚB, ii) type I interferon through the transcription factors IRF3/7, and iii) IL1-β, through the caspase 1 activation, all of which contribute directly to obesity mediated-metaflammation. Figure was created with biorender.com.

Multiple recent studies have reported that obese individuals or mice exposed to a high-fat diet (HFD) show elevated levels of circulatory cell-free DNA (cfDNa) (14, 15). Sources of cfDNA vary among obese patients and mouse models of obesity (**Figure 1**). Murine hepatocytes from livers affected by NAFLD were shown to acquire the potential to release mitochondrial DNA (mtDNA) in microparticles (MPs) (15). This MP-associated mtDNA was significantly increased in the plasma of obese patients, particularly in those who had active NAFLD (15). Obesity was also reported to induce neutrophils and MΦ to release extracellular traps (ET), which are composed of NA and antimicrobial peptides (16). Such ET were more abundant in the WAT of obese mice compared to lean mice and showed potent inflammatory properties *in vitro* (16). Importantly, bone marrow-derived myeloid cells from obese mice fed HFD were more susceptible to extrude ET containing DNA upon *in vitro* stimulation, indicating that obesity may systemically boost the potential of myeloid cells to release ET (16). Finally, oxidative stress, hypoxia, inflammation and aberrant adipogenesis that occur during obesity lead to heightened cell death of adipocytes that release both their genomic (g)-DNA and mtDNA, and thus contribute to the systemic accumulation of cfDNA (17). Accordingly, Nishimoto et al. observed *in vitro* that epididymal fat from mice fed HFD constitutively released more cfDNA than the fat from animals on normal diet (14). Furthermore, explant of WAT from obese individuals released elevated levels of self-DNA in culture supernatants (18). The DNA released by WAT explants was associated with high mobility group box 1 (HMGB1), a nuclear protein that was previously shown to be an endogenous DAMP and increases the inflammatory potential of self-DNA (19). Importantly, systemic levels of these cfDNA and HMGB1 positively correlated with the severity of metabolic syndrome induced by obesity including the extent of visceral fat tissue, insulin resistance, and liver injury (14, 15, 20, 21).

In addition to their elevated quantity, the quality of cfDNA is also affected by obesity. Obesity-mediated inflammation induces an accrual of reactive oxygen species (ROS) that cause DNA oxidation (DNA-OH) (22). Guanosines are the targets of ROS-mediated oxidation and the 8 hydroxydeoxyguanine (8-OHdG) represent the main DNA oxidation marker. Garcia-Martinez et al. showed that the vast majority of mtDNA contained in the circulatory MPs of obese individuals affected by NAFLD contain 8-OHdG reflecting its oxidized nature (15). Such oxidized forms of mtDNA from obese individuals exhibit an elevated potential to stimulate DNA sensing PRR (15) as previously reported in autoimmune contexts (23, 24), likely due to its protection form the degradation by nucleases (25).

Obesity not only modulates systemic levels of self-DNA, but also causes bacterial DNA leakage from the gastrointestinal tract. HFD was shown to modulate intestinal permeability and to contribute *via* the portal circulation to the transport of bacterial DNA to the liver where it ultimately activates inflammatory responses and promotes NAFLD development (26). However, the impact of bacterial DNA to NAFLD pathogenesis was only observed in inflammasome-deficient animals, which present a major intestinal dysbiosis (26). Thus, these observations may explain the susceptibility of certain individuals to obesity-mediated pathogenesis rather than represent a general mechanism of action of obesity.

Overall, obesity appears to induce the accumulation of NA originating from various sources and such NA participate in obesity-mediated pathogenesis (**Figure 1**). Not only obesity increases the quantity of NA, but it also affects their overall quality. Various forms of cfDNA with an enhanced immunogenic potential are preferentially detected in obese individuals, including MPs associated-DNA, ET associated-DNA, HMGB1 bound-DNA and oxidized-DNA. While different sources and forms of DNA were reported to accumulate during obesity, their individual contribution to complications induced by obesity remain unknown. Besides, whether self-RNA accumulate in obese patients and in which forms, require further investigation.

Multiple safeguard mechanisms are involved in the disposal of dying cells and their NA, preventing NA ability to activate inflammatory immune responses. These protective mechanisms include the clearance of apoptotic cells by phagocytes (efferocytosis) (27) and the digestion of extracellular DNA by circulatory deoxyribonucleases (DNASEs) (28). It is becoming clear that obesity impairs phagocytes ability to clear apoptotic cells by affecting lipid composition of cell membranes and their expression of scavenger receptors (29, 30). Furthermore, HFD in mice was reported to reduce the overall circulatory DNASE activity (16). However, treatment of obese mice with recombinant DNASE1 did not affect the development of metabolic syndrome (16). This absence of therapeutic potential of DNASE1 may be due to soluble mediators present in obese mice that block DNASE1 function and/or to its inability to clear all sources of pathogenic DNA that accumulate during obesity. Indeed, DNASE1 is only capable of digesting “naked” DNA in the extracellular space, so exploring the role of other circulatory DNASES in the regulation of obesity-mediated pathogenesis is essential. It will be particularly relevant to address the function of DNASE1L3 in this context, since it is specifically expressed in immune cells infiltrating metabolic tissues and digest both MP-associated DNA (31, 32) and neutrophil ET-associated DNA (33), the two main forms of DNA that accumulate during obesity. Overall, in addition to an accrual endogenous DNA release, obesity also impair safeguard mechanisms that are involved in their disposal, and these processes together participate in the accumulation of self-DNA in obese individuals.

## Nucleic Acid Sensing Pathways Involved in Obesity-Mediated Metaflammation

The sensing of NA is operated by two major classes of PRRs, including endolysosomal and cytosolic NA sensors (**Figure 1**), which in response to stimulation trigger the production of inflammatory cytokines such as type I interferons (IFN-I), IL-1β, and TNF-α, playing a crucial role in obesity-mediated metaflammation (13).

TLRs comprise all-known NA sensing endolysosomal PRR. Among them, TLR9 is specialized in the recognition of DNA, and its deficiency was recently reported to protect from the development of metabolic syndrome induced by HFD. Indeed, *Tlr9-*deficient mice displayed a reduction in WAT and liver inflammation and improved insulin sensitivity compared to wild-type mice upon HFD (14, 16). Injection of the TLR9 ligand CpG DNA (ODN2395) in mice increased fasting glucose levels and immune cell infiltration in WAT and liver, indicating that even in the absence of obesity TLR9 activation leads to metabolic deregulations (16). Conversely, administration of TLR9 antagonist (iODN2088) to HFD-fed mice attenuated metabolic tissue inflammation, improved glucose metabolism (14), and ameliorated manifestation of liver steatosis (15), confirming the crucial role of TLR9 in obesity-mediated pathogenesis. This aberrant activation of TLR9 during obesity was shown to be mediated by the recognition of self-DNA released by dying adipocytes, MPs loaded with mtDNA as well as ET (14–16, 18). TLR3 and TLR7 are also endolysosomal PRRs, but they are involved in the recognition of ds and ssRNA, respectively. The contribution of TLR3 and TLR7 to obesity-mediated pathogenesis was established when their deficiencies were shown to restore glucose tolerance, decrease metabolic inflammation and ameliorate NAFLD in HFD fed animals (16, 34, 35). TLR8, which is also a sensor of ssRNA, showed an increased expression in MΦ infiltrating the WAT of obese patients with or without T2D, and TLR8 expression significantly correlated with disease severity and metabolic tissue inflammation (36). Although TLR8 is unresponsive to ssRNA in mice, its deficiency induced mild metabolic abnormalities and increased the liver inflammation in HFD fed mice (37). This observation was mainly due to increased TLR7 expression in TLR8 knock-out mice (37). The net contribution of TLR8 in obesity thus requires further investigation particularly in transgenic mice expressing human TLR8 (38). While RNA-sensing TLRs seem to play a role in obesity, the origins of the pathogenic RNA, its form and its regulation during obesity remain unknown. Most TLRs signal through the adaptor molecule myeloid differentiation primary response (MyD)-88 with the exception of TLR3, which transduces signaling *via* TIR-domain-containing adapter-inducing interferon-β (TRIF). MyD88 activation leads to the production of inflammatory cytokines through the activation of nuclear factor kappa-B (NF-ΚB) which was shown to play a critical role in obesity-mediated inflammation (12). Alternatively, endolysosomal TLR3 signaling activates the transcription factor interferon regulatory factor (IRF)-3 *via* TRIF and TLR7-9 activate IRF7 *via* MyD88 (**Figure 1**). Both IRF3 and IRF7 are involved in the induction of IFN-I production (39), which has also been reported to play an important role in obesity-mediated metabolic syndromes. Specific deletion of *Irf7* (40) or IFN-I receptor (*Ifnar*) improved obesity-mediated inflammation and insulin resistance (41). In contrast, the role of IRF3 in obesity is more controversial. *Irf3* deficiency was shown to alleviate adipose tissue inflammation and insulin resistance in HFD-fed mice (42), yet it also exacerbated the development of NAFLD induced by HFD (43). Therefore, IRF3-mediated IFN-I production may have tissue-specific functions and play a protective role in liver pathology induced by obesity.

Cytosolic NA sensing PRRs include Rig-I like receptors (RLR) that are specialized in the sensing of RNA, the DNA-sensor cyclic GMP-AMP Synthase (cGAS), and the absent in melanoma (AIM)-2 inflammasome that specifically detects dsDNA. While RLR function in obesity-mediated inflammation is poorly studied, a role for cytosolic DNA sensing pathways is being defined (44). The DNA sensor cGAS signals through stimulator of IFN genes (STING) to induce the production of IFN-I in an IRF3-dependent manner and the production of inflammatory cytokines through NF-ΚB (45) (**Figure 1**). STING expression was reported to be upregulated in the livers of NASH patients compared to healthy controls (46). Furthermore, liver inflammation and steatosis was significantly improved in mice deficient for *Tmem173* (gene encoding STING) that were fed a HFD (46, 47). Despite the effect on liver pathology, *Tmem173* deficiency did not show any impact on glucose metabolism in obese mice (47). The activation of cGAS-STING pathway upon HFD was shown to be mediated by mtDNA released by hepatocytes, which *via* NF-ΚB leads to preferential production of TNF-α and IL-6 (47). IFN-I was not detected in the liver of HFD mice or in supernatants of liver MΦ (Kupffer cells) stimulated with mtDNA isolated from hepatocytes (46, 47), suggesting that the cGAS pathway contributes to NASH independently of IFN-I. These observations are in accordance with the previously discussed literature indicating that IRF3 may protect against NASH development induced by obesity (43). More recently, obesity induced either by HFD or genetically by the deficiency of the leptin receptor (*db/db* mice) was shown to promote the accumulation of mtDNA and the activation of the cGAS pathway in adipocytes (48). The accumulation of mtDNA into the cytosol of adipocytes was suggested to be due to the inhibition of the disulfide-bond A oxidoreductase-like protein (DsbA-L), which is a mitochondrial matrix chaperone. Accordingly, fat-specific deficiency of DsbA-L aggravated the weight gain and glucose intolerance of HFD-fed mice while WAT-specific overexpression of DsbA-L protected mice from obesity-induced inflammation and insulin resistance (48). These results suggest that beyond NASH cGAS may contribute to obesity-induced metabolic syndromes. However, it remains to be explored whether the impact of DsbA-L deficiency is dependent on the cGAS-STING pathway *in vivo*.

Finally, various members of the inflammasome play a crucial role in obesity-mediated pathogenesis (11). Among inflammasomes, DNA is primarily detected by the AIM-2 inflammasome. AIM-2 triggers caspase-1 activation which is essential for the cleavage of pro-IL-1β and pro-IL-18 into their mature and biologically active forms. Recently circulatory mtDNA isolated from patients with T2D was reported to contribute to AIM-2 inflammasome-dependent caspase-1 activation and IL-1β and IL-18 secretion by MΦ (49) (**Figure 1**). However, the *in vivo* role of AIM-2 in obesity remains controversial. Gong et al. have observed spontaneous obesity, impaired glucose metabolism, and increased WAT inflammation in *Aim-2* deficient mice (50). These results are not only intriguing but also consistent with the observation that the inflammasome has a dual role in obesity, contributing to obesity-mediated inflammation *via* IL-1β (51) and preventing its negative impact by the production of IL-18 (52). Finally, the NLRP3 inflammasome was previously reported to promote obesity-mediated pathogenesis upon its activation by intracellular ceramide (53). Interestingly, oxidized mtDNA (54) and oxidized DNA originating from NETs (55) were also shown to activate the NLRP3 inflammasome (**Figure 1**). Therefore, such DNA that accumulates during obesity is likely to contribute to obesity-mediated pathogenesis though the activation of NLRP3 as well.

## Cellular Players Involved in Nucleic Acid Detection During Metaflammation

Innate immune cells, in particular DC and MΦ, play a crucial role in the recognition of NA and the ensuing production of inflammatory cytokines. While innate immune cells have been shown to participate in obesity-associated inflammation, the contribution of their ability to recognize NA to this process in only beginning to be understood.

It was recently proposed that NA originating from ETs, which accumulate in obese mice, directly activate the production of proinflammatory cytokines by MΦ *via* TLR-7 and TLR-9 (16). Targeted deletion of *Tlr9* in MΦ using *LysM*-Cre protected mice from the development of NAFLD induced by HFD (15). Furthermore, the transfer of STING-deficient bone marrow cells ameliorated HFD induced NAFLD in bone marrow chimeras. In view of these results the authors suggested that the activation of the cGAS-STING pathway in Kupffer cells was crucial for NAFLD pathogenesis (46). However, Kupffer cells are radioresistant and the contribution of bone marrow cells for their replenishment is low (56), therefore it is more likely that cGAS-STING pathway is required in another cell type of hematopoietic origin for the development of NAFLD.

DC contribution to obesity-mediated pathogenesis was previously documented (57); however, specific deletion of NA sensing pathway in DCs during obesity has not yet been investigated. TLR9 is broadly expressed among immune cells in mice, but only in plasmacytoid dendritic cells (pDCs) and B cells in humans (18). Importantly, pDCs are a subset of DCs that are specialized in the production of IFN-I upon sensing of microbial and self-NA (58). pDCs were recently shown to be recruited to the WAT of obese mice and individuals (18, 57, 59) and their infiltration into the WAT correlated with obesity-associated insulin resistance (59). Importantly, DNA-complexed with HMGB1 released by human adipocytes stimulated pDC production of IFN-I in a TLR9-dependent manner (59). Moreover, specific ablation of pDCs ameliorated obesity-associated metabolic syndrome and insulin resistance *in vivo* (41). However, whether pDC-specific expression of TLR9 and production of IFN-I are directly involved in obesity-mediated metabolic syndromes *in vivo* require further investigation.

Despite the systemic accumulation of cfDNA in obese individuals, its ability to activate inflammatory responses in circulatory leukocytes remains poorly documented. It appears that cfDNA primarily activates innate immune cells within metabolic tissues (18, 46). Inflammatory cytokines produced in response to such stimulation are then redistributed systematically upon their release in the circulation. Given the potential of inflammatory cytokines to activate circulatory leukocytes and to further fuel systemic inflammation, it is quite difficult to distinguish the stimulatory impact on circulatory leukocytes of inflammatory cytokines from the one of cfDNA. Nevertheless, *in vitro* stimulation of a monocytic cell line with healthy individuals’ plasma was recently shown modulate their innate immune functions, mostly through cfDNA (60). Given that such cfDNA accumulate during obesity, its ability to directly stimulate circulatory leucocytes and ultimately contribute to obesity-mediated metaflammation requires further investigation.

The cGAS-STING pathway is ubiquitously expressed. It was recently reported that obesity induces the accumulation of mtDNA directly in adipocytes, which ultimately activates the cGAS-STING pathway. In response to such stimulation, these adipocytes produce inflammatory cytokines including IFN-I, contributing to the overall metaflammation induced by obesity (48). These observations indicate that obesity-mediated metaflammation is driven by not only immune cells but also adipocytes.

IFN-I that is produced in response to self-DNA sensing, induces the expression of a plethora of IFN-stimulated genes (ISGs) whose expression was reported to be associated with adipose tissue and systemic insulin resistance in obese patients (18). This pathogenic role of IFN-I in obesity is likely due to its ability to (i) polarize adipose tissue MΦ toward a proinflammatory M1 phenotype (18), (ii) activate innate and adaptive immune responses (61, 62) and (iii) to amplify adipocytes’ cell-intrinsic inflammatory capacity (63). Apart from the indirect impact on inflammatory processes, IFN-I was also shown to directly interfere with insulin signaling in metabolically active tissues, particularly in adipocytes (64) and hepatocytes (65). Therefore IFN-I induced in response to the aberrant sensing of self-NA by immune and non-immune cells clearly contributes to obesity-mediated pathogenesis.

## Future Directions and Therapeutic Avenues

In addition to their contribution to inflammatory and in autoimmune diseases (13), it is now clear that NA accumulation and its sensing by various PRRs contribute to the development of metabolic syndrome induced by obesity (as summarized in **Figure 1**). The identification of these novel pathways has opened new therapeutic options. Indeed, Hydoxychloroquine, which blocks endosomal acidification and thus endolysosomal TLR function (66), may have beneficial effects on insulin resistance in obese non-diabetic individuals (67) and prevent the onset of diabetes in patients with autoimmune diseases (rheumatoid arthritis, SLE, psoriasis…) (68, 69). Furthermore, specific antagonists of TLR7,9 and STING, which are in early-phase trials for SLE (70) and various interferonopathies (71), respectively, also show promise for obesity treatment given their therapeutic potency in mouse models of the disease. Similarly, monoclonal antibodies targeting IFN-α (sifalimumab) (72) or IFNAR1 (anifrolumab) (73) and specific ablation of pDCs, which are the main IFN-I producing cells (74), may also have therapeutic value in obese individuals in addition to SLE patients in view of the importance of the pDC-IFN-I axis in obesity. Despite these advances, the specific mechanisms through which HFD and obesity modulate the abundance of nucleic acids remain poorly understood. Understanding these mechanisms will provide a better understanding of the initiation and progression of obesity pathogenesis as well as novel potential therapeutic approaches.

## Author Contributions

All authors contributed to the article and approved the submitted version. AF and VS wrote the manuscript.

## Funding

This work was supported by research grant from the Agence Nationale de la Recherche (ANR JCJC DOMINOS) (VS), the IdEx Junior Chair program from the University of Bordeaux (VS), the Indo-French Centre for the Promotion of Advanced Research grant (IFCPAR) (VS, DG) and by the Fondation Bordeaux Université (VS, AF).

## Conflict of Interest

The authors declare that the research was conducted in the absence of any commercial or financial relationships that could be construed as a potential conflict of interest.

## Abbreviations

WAT, White adipose tissue; NA, Nucleic Acids; NAFLD, Non-Alcoholic Fatty Liver Disease; NASH, Non-Alcoholic Steatohepatitis; HFD, High Fat Diet; PRR, pathogen recognition receptors; PAMP, Pathogen-associated molecular pattern; DAMP, Danger-associated molecular pattern; NETosis, cell death by Neutrophil Extracellular Traps; gDNA, Genomic Desoxyribonucleic Acid; mtDNA, mitochondrial DNA; CfDNA, Cell-free DNA; MP, Microparticles; HMGB1, high–mobility group box 1; RNA, Ribonucleic Acid; TLR, Toll-Like-Receptor; AIM2, absent in melanoma 2; cGAS, cyclic GMP–AMP synthase; STING, stimulator of interferon genes; NLRP3, NLR family pyrin domain containing 3; TRIF, TIR-domain-containing adapter-inducing interferon-β; MyD88, myeloid differentiation protein 88; NF-κB, nuclear factor-kappa B; IRFs, Interferon Regulatory Factors; IL-, Interleukin; TNF, Tumor Necrosis Factor; IFN, Interferon; IFNAR, IFN α/β receptor; DC, Dendritic Cells; pDC, Plasmacytoid dendritic cells; Mφ, Macrophages.
